# Notes and News

**Published:** 1894-09-29

**Authors:** 


### Notes and News.

Collected and Communicated.

The third annual congress of the Society for the Prevention of Cruelty to Children will be held in Birmingham on October 23rd. A list of important speakers is announced.

Medicine at Bordeaux is receiving considerable State encouragement at present. The town has just received a grant of 500 francs for its ambulance service, the same sum for its medical and chirurgical society, and 100,000 francs for the enlargement of the Hospice de Pellegrin. A thousand francs has also been voted towards the expenses of the International Congress for the Protection of Children, to be held in July, 1895.

In spite of the gloomy, cold, and variable weather which has been almost universal this summer, the health reports from most parts of England in the middle of September are better than usual. In Paris the report is still more favourable. Health in that city appears to have been better than for over twelve years. We have heard a good deal of late of Paris, as showing a great tendency to an increase of typhoid. It must encourage those who feel any fear of unusual risk when visiting the French capital, to know that only three deaths from this disease during the first week in September were recorded.

It would seem that the estimation as to what are the "best interests" of a man may differ much. A workman at Buckingham Palace received an injury to his eyes, and was received into the Middlesex Hospital. The Queen entrusted £10 to the chairman of the hospital, to be devoted to his "best interests." It is stated that the man has been away from the hospital for nearly a month, and that the authorities have not yet decided that he is to have the money. He, in the meanwhile, states himself to be in need of aid, and has applied to the police-court to assist him to obtain the £10. The man's case has received the most careful consideration at the hands of the hospital authorities, and under these circumstances it would be a mistake to conclude that he has been hardly used.

The students' dinner season at the hospitals is now approaching. At the London, the dinner is announced to take place on Monday, October 1st; also at the Middlesex Hospital and St. Mary's on the same day.

There is an art in opening the hearts of classes or individuals towards generous giving. Very unjustly, it was pointed out in the press that the titled classes were not as generous in their support of charity as others of the community. That sympathy and interest must be secured before a donation is forthcoming is the usual experience of those who collect money for charitable objects. The Victoria Hospital for Children at Chelsea is at present a striking example that the upper classes are not backward in giving. Anyone who would verify this should glance down the subscription list of the "Infant Prince Fund" raised to commemorate the birth of the Heir to the Throne. Already £700 has been contributed to the fund, for the benefit of the hospital.

The centenary of the Sunderland Infirmary has been celebrated by the addition of a new wing to the Heatherdene Convalescent Home, Lancaster. The foundation-stone was laid last week in the presence of a large company. Little more than two years ago the home itself was opened. It has since received over 400 women and children within its walls. The new wing will extend its advantages to male patients. Two acres of ground are also included in the extension, to purchase which, and build and furnish the new wing, £4,500 is required. More than half this sum has already been subscribed. The wing will comprise day, dining, and sleeping rooms, together with offices and accommodation for servants, which will render it practically self-contained. At the foundation-stone ceremony Mr. Lawson, one of the workmen's governors, handed a cheque to the Mayor for the institution, amounting to £105, the proceeds of the demonstration and sports held by the working men in celebration of the centenary of the Sunderland Infirmary.

The aim of all complete education must be to render men fit to meet the vicissitudes of life. Education in England and elsewhere is becoming increasingly practical. Physical training, especially in large towns, is a difficulty, whilst it is an imperative necessity for the making of capable and healthy citizens. Any advance in this direction should be encouraged, and useful lessons duly learnt. We have just received an invitation which proves that the Jewish community are alive to the advantage of physical training, and determined to be in the vanguard of progress in this matter. We are invited to attend a swimming competition of the Jewish Schools of Stepney. This competition has been established for five years, and during the last two years swimming has also been taught to the girls. No one can for a moment question the advantage of encouraging a love not only of the exercise, but of water to the inhabitants of sooty London, and if it be held that Government has done enough at present for the education of the masses, there is at any rate an admirable opening for the philanthropist to render a public service by instituting swimming instruction at one or a number of schools, free, or at a small cost to the learners.

